# Gonorrhoea diagnoses in a network of STI clinics in Spain during the period 2006–2010: differences by sex and transmission route

**DOI:** 10.1186/1471-2458-13-1093

**Published:** 2013-11-25

**Authors:** Asuncion Diaz, Cesar Garriga, Jose Antonio Varela, Elisa Fernández, Isabel Sanz, Josep Boronat, Fuensanta Gual, Concepcion Colomo, Josefina López de Munain, Valentin Esteban, Maria Luisa Junquera, Blanca Martínez, Isabel Pueyo, Justo Suárez, Maria Jesus Barberá, Maider Arando, Jose Manuel Ureña, Mercedes Diez

**Affiliations:** 1 Instituto de Salud Carlos III, Área de Epidemiología del VIH y comportamientos de riesgo, Centro Nacional de Epidemiologia, C/ Monforte de Lemos, 5, 28029 Madrid, Spain; 2 Centro ETS, Gijón, Spain; 3 Centro de Información y Prevención del Sida, Alicante, Spain; 4 Plan del Sida del País Vasco, San Sebastián, Spain; 5 Unidad ITS, CAP Tarragonès, Tarragona, Spain; 6 Comité de Apoyo a las Trabajadoras del sexo (CATS), Murcia, Spain; 7 Programa de Prevención del Sida y ETS, Madrid, Spain; 8 Unidad ETS, Enfermedades Infecciosas, Hospital de Basurto, Bilbao, Spain; 9 Servicio Microbiología Clínica, Hospital Basurto, Bilbao, Spain; 10 Unidad de ETS, Hospital Monte Naranco, Oviedo, Spain; 11 Unidad de Promoción y Apoyo a la Salud (UPAS), Málaga, Spain; 12 Centro ETS, Sevilla, Spain; 13 Centro ETS, Algeciras, Spain; 14 Unidad de ITS. Programa especial de enfermedades infecciosas Vall d’Hebron-Drassanes, Hospital Universitario Vall d’Hebron, Barcelona, Spain; 15 Centro de ETS y Orientación Sexual, Granada, Spain; 16 Plan Nacional sobre el Sida. S.G. de Promoción de la Salud y Epidemiología. Ministerio de Sanidad, Servicios Sociales e Igualdad, Madrid, Spain; 17 CIBER de Epidemiología y Salud Pública (CIBERESP). Instituto de Salud Carlos III, Madrid, Spain

**Keywords:** Gonorrhoea, Genitourinary medicine services, STI, HIV, Core populations

## Abstract

**Background:**

Gonorrhoea infection is one of the most common bacterial sexually transmitted infections and an important cause of morbidity and serious complications. The objectives of this paper are: a) to describe gonorrhoea cases diagnosed in a network of 15 (out of 16) STI clinics in Spain during 2006–2010; b) to analyse differences among men who have sex with men (MSM), men who have sex exclusively with women (MSW) and women; and c) to evaluate factors associated to with HIV co-infection.

**Methods:**

All gonorrhoea cases diagnosed in the network were included (25.7% of total cases notified in Spain). Data were collected by clinical staff. Descriptive/bivariate analyses were carried out stratifying by sex and transmission category; association and trends were evaluated using the chi-square test. Factors associated with HIV co-infection were estimated using a logistic regression model.

**Results:**

2385 cases were included: 55.3% among MSM, 31.3% among MSW and 13.3% among females; cases among MSM increased from 55.8% in 2006 to 62.9% in 2010 while no trends were found among the other two groups.

Most MSM cases were Spaniards (72%), aged 25–34 years (46%), 49% reported previous STI and 25% concurrent STI (excluding HIV); casual partners were the commonest source of infection, and 21% of cases had rectal gonorrhoea. MSW cases did not differ from MSM by age, origin or source of infection, but frequencies of prior or concurrent STI were lower. Female cases were younger than male, were mostly foreigners (58%), and 41% were sex workers; concurrent STI (other than HIV) were diagnosed in 30%; 20.4% had symptoms (72.5% and 89.2% in MSM and MSW), and pharyngeal location was present in 30%.

HIV co-infection was highest in MSM (20.9%). Co-infection was associated with age > 35 years, low educational level, being Western European or Latin-American, being MSM, having previous or concurrent STI and reporting contact with an HIV-infected partner; it was inversely associated with female sex.

**Conclusion:**

Differences by sex, transmission route and origin should be considered when implementing care and preventive programmes for gonorrhoea, and MSM are a priority group for intervention, in particular HIV-infected MSM.

## Background

Infection with *Neisseria gonorrhoeae* is one of the most common bacterial sexually transmitted infections (STI), and an important cause of morbidity and serious complications, such as pelvic inflammatory disease, infertility and ectopic pregnancy in women and epididymo-orchitis in men [1]. Additionally, gonorrhoea infection may contribute to facilitating HIV transmission [2].

Since the late 1990s, several Western European countries have reported an increase in the number of gonorrhoea diagnoses [3]. Men, people older than 25 years and men who have sex with men (MSM) are overrepresented among gonorrhoea cases [3,4]. The emergence of strains resistant to first-line antimicrobials adds complexity to patients´ treatment and threatens disease control [5].

In Spain, nationwide surveillance of the total number of gonorrhoea diagnoses has been in operation since 1982. In 2010 the reported rate of gonorrhoea infection for the whole country was 5.1 per 100 000 population [6], which is lower than the 10.4/100 000 overall rate notified in the EU/EEA in the same year, and lower than the rates of other West European countries, although comparisons are difficult due to the heterogeneity of surveillance systems [3]. Notified rates currently show an increasing trend, but the rise started later than in other countries: during the period 1995–2001 rates decreased sharply, from 11.7/100 000 to 2.0/100 000, but in 2002 the trend changed and the incidence has steadily increased to a rate of 5.1/100 000 in 2011 [6]. Unfortunately, the current national surveillance system for gonorrhoea does not include the collection of individual data on cases so it is not possible to know which groups are most affected by the increase.

In addition to the countrywide surveillance system, beginning in January 2006, a sentinel surveillance project was started in a network of clinics specialising in STI, the EPI-ITS Network. The project objective was to collect detailed clinical and epidemiological data, including HIV co-infection, in all gonorrhoea and syphilis cases diagnosed in the EPI-ITS Network. Yearly reports as well as a paper with results from this project have been published previously [7,8].

The objectives of this paper are: a) to describe the characteristics of gonorrhoea cases diagnosed in a network of 15 clinics from 2006 to 2010; b) to analyse differences among cases who were MSM, men who have sex exclusively with women (MSW) and women, respectively; and c) to investigate factors associated with HIV co-infection.

## Methods

All gonorrhoea cases identified between January 2006 and December 2010 in the EPI-ITS Network were included in the study. The network includes 15 STI clinics, located in 14 of Spain’s most populated cities (Madrid, Barcelona, Seville, Malaga, Bilbao (two clinics), Granada, Algeciras, Oviedo, Gijon, San Sebastian, Tarragona, Cartagena, Murcia and Alicante). These are public, low threshold clinics with a long tradition of attending patients belonging to STI “core” populations and their sexual partners (i.e. “bridge” populations); according to World Health Organization (WHO) [9], “core” populations include: MSM, sex workers and their clients, immigrants and any person with highly-risky behaviors for STI. Services are provided free of charge, even for illegal immigrants, and every effort is made to maximize accessibility (including, in most clinics, providing care anonymously if the clients so wish). Participation in the project is voluntary but, to our knowledge, more than 90% of the STI clinics in Spain (15 out of 16) belong to this network.

Cases diagnosed in the network represented 25.7% of the total number of gonorrhoea cases notified to the population surveillance system in Spain during the study period (27.2% in 2006, 22.6% in 2007, 24.5% in 2008, 29.7% in 2009 and 24.7% in 2010). In addition to STI clinics, gonorrhoea cases in Spain can be diagnosed in a variety of settings, including primary care centres, gynaecological and dermatological clinics, and those providing family planning services, both in the public and private sector.

Socio-demographic (age, sex, educational level, region of birth) and clinical variables (HIV status, history of previous STI, other concurrent STI, anatomical location, reason for attending), as well as information on the circumstances of infection (possible country of infection, source of infection), were collected from the clinical records by the clinical staff. A common questionnaire developed for the project was used.

Gonorrhoea cases included in this analysis were identified using the European definition: i.e. “a *gonorrohea confirmed case is any person meeting at least one of the laboratory criteria* (*isolation of Neisseria gonorrhoeae from a clinical specimen*, *detection of Neisseria gonorrhoeae nucleic acid in a clinical specimen*, *demonstration of Neisseria gonorrhoeae by a non amplified nucleic acid probe test in a clinical specimen or microscopic detection of intracellular gram negative diploccocci in an urethral male specimen*) *with or without symptoms*” [10]. To assign region of birth, the WHO-Europe definition was used. A case was considered a sex worker if he/she reported to have been paid for sex. Concurrent STI was defined as any STI diagnosed by the physician caring for the case simultaneously to gonorrhoea; the following categories were used for data collection: a) *Chlamydia trachomatis* infection (including lymphogranuloma venereum); b) genital warts; c) herpes virus; d) syphilis; e) trichomoniasis; d) molluscum contagiosum; e) other (specify). Routine clinical practice for STI care and diagnosis in Spain includes offering HIV testing, as well as screening for other STIs, to any STI patient [11].

Data were collected in the context of sentinel surveillance, as described in the Spanish Multisectorial Plan on HIV/AIDS, 2008–2012; therefore, neither ethical approval nor informed consent was required (Ley 33/2011, de 4 de octubre, General de Salud Pública). The only identifier included in the questionnaire was an alphanumeric code exclusively used for quality control after which data were treated and analysed anonymously. The ethical principles of the Helsinki Declaration of 1964 (revised by the World Medical Organization in Edinburgh, 2000) were followed, and to comply with the requirements of the Organic Law 15/1999 of Data Protection in Spain, the database was registered in the Spanish Data Protection Agency (Registry number 2080910068).

Cases with missing information, either on gender or on gender of their sexual partners (n = 20), were excluded from the global analysis. Frequency distributions for each categorical variable, stratified by sex and transmission route (MSW, heterosexual females and MSM), were calculated, as well as the median and 25^th^ and 75^th^ percentiles (P_25_-P_75_) for age. To evaluate the association between categorical variables the chi-squared or Fisher’s tests were used, and the U Mann–Whitney test for age. Trends were evaluated using the chi-square for trend.

HIV/gonorrhoea co-infection was calculated and a logistic regression model was fitted to identify factors associated with co-infection. The adjusted odds ratio and its 95% confidence interval (AOR; 95% CI) was the measure of association.

## Results

From 2006 to 2010, a total of 2385 gonorrhoea cases were identified in the participating centres. Of these, 1320 (55.3%) occurred among MSM, 747 (31.3%) among MSW and 318 (13.3%) among heterosexual females; twenty cases with missing information, either on gender or on gender of their sexual partners, were excluded from the analysis.

**Figure 1:**
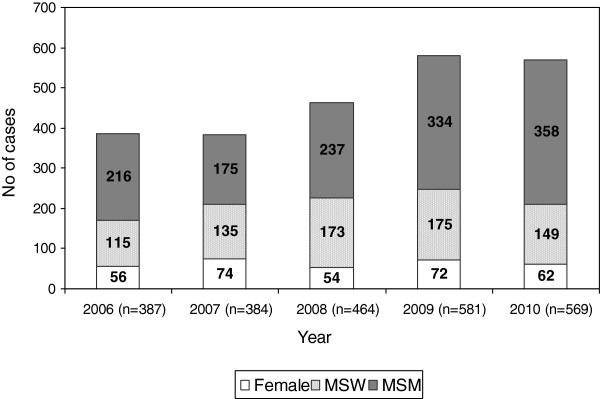
**Gonorrhoea cases diagnosed in 15 STI clinics in Spain from 2006 to 2010, by year of diagnosis, sex and transmission route.** *MSW: Men who have sex exclusively with women; MSM: Men who have sex with men.

The number of cases collected increased from 387 in 2006 to 569 in 2010 (Figure 1). More than half of the annual diagnoses occurred in MSM (range: 45.6%-62.9%), and an increasing trend was found in the proportion of MSM diagnosed with gonorrhoea during the study period (from 55.8% in 2006 to 62.9% in 2010) (p < 0.05). No trends were found among the other two groups.

### Socio-demographic characteristics

Median age of cases was 29 years (IQR: 24–36), lower in females than in MSW (27.0 years (P_25_-P_75_: 22–33) vs. 30.0 years (P_25_-P_75_: 25–36), respectively) (p < 0.05), while no difference was found between MSM and MSW. Only among the latter, the percentage in the age group of 25–34 years increased from 36.3% in 2006 to 47.3% in 2010.

More MSM than MSW had completed secondary/university education (p < 0.05) without differences between MSW and women. By region of birth, MSM and MSW were mostly Spaniards while females were mostly born outside Spain. The commonest region of birth among foreigners was Latin America, but the second region differed across all groups: for MSM, this region was Western Europe, for MSW it was Africa, and for women, Central/Eastern Europe (Table 1). In the study period an increasing trend of diagnosed gonorrhoea was observed in both Latin-American (from 13.4% in 2006 to 16.5% in 2010) and Western European MSM (from 5.6% in 2006 to 8.7% in 2010), whereas no trend was observed in the other groups.

**Table 1:** Characteristics of gonorrhoea cases, by sex and transmission route

	**Female (n = 318)**		**Men who have sex exclusively with women (MSW) (n = 747)**		**Men who have sex with men (MSM) (n = 1320)**		**Total (n = 2385)**	
	**n**	**(%)**	**n**	**(%)**	**n**	**(%)**	**n**	**(%)**
**Age group (years)**								
<20	28	(8.8)	49	(6.6)	41	(3.1)	118	(4.9)
20-24	92	(28.9)	135	(18.1)	253	(19.2)	480	(20.1)
25-34	130	(40.9)	341	(45.6)	612	(46.4)	1083	(45.4)
35-44	43	(13.5)	146	(19.5)	319	(24.2)	508	(21.3)
≥ 45	24	(7.5)	70	(9.4)	93	(7.0)	187	(7.8)
Unknown	1	(0.3)	6	(0.8)	2	(0.2)	9	(0.4)
**Educational level completed**								
Illiteracy	5	(1.6)	15	(2.0)	5	(0.4)	25	(1.0)
Primary education	83	(26.1)	214	(28.7)	149	(11.3)	446	(18.7)
Secondary education	83	(26.1)	195	(26.1)	360	(27.3)	638	(26.8)
University education	36	(11.3)	86	(11.5)	434	(32.9)	556	(23.3)
Unknown	111	(34.9)	237	(31.7)	372	(28.2)	720	(30.2)
**Region of birth**								
Spain	134	(42.1)	513	(68.7)	950	(72.0)	1597	(67.0)
Western Europe	5	(1.6)	14	(1.9)	93	(7.0)	112	(4.7)
Central/Eastern Europe	32	(10.1)	41	(5.5)	23	(1.7)	96	(4.0)
Latin America	127	(39.9)	83	(11.1)	209	(15.8)	419	(17.6)
North Africa	5	(1.6)	51	(6.8)	16	(1.2)	72	(3.0)
Sub-Saharan Africa	10	(3.1)	23	(3.1)	2	(0.2)	35	(1.5)
Other/Unknown	5	(1.6)	22	(2.9)	27	(2.0)	54	(2.3)
**Previous STI (other than HIV)**	59	(18.6)	169	(22.6)	640	(48.5)	868	(36.4)
HIV diagnosed*	3	(1.0)	15	(2.3)	249	(20.9)	267	(12.4)
**Concurrent STI (other than HIV)**	95	(29.9)	138	(18.5)	326	(24.7)	559	(23.4)
One	87	(27.4)	126	(16.9)	293	(22.2)	506	(21.2)
More than one	8	(2.5)	12	(1.6)	33	(2.5)	53	(2.2)
**Reason for attending the clinic**								
Symptoms	65	(20.4)	666	(89.2)	957	(72.5)	1688	(70.8)
Screening	92	(28.9)	15	(2.0)	132	(10.0)	239	(10.0)
Partner notification	113	(35.5)	11	(1.5)	118	(8.9)	242	(10.1)
Unknown	48	(15.1)	55	(7.4)	113	(8.6)	216	(9.1)
**Anatomical location**								
Urethra	3	(0.9)	694	(92.9)	769	(58.3)	1466	(61.5)
Rectum	10	(3.1)	0	(0.0)	278	(21.1)	288	(12.1)
Pharynx	94	(29.6)	7	(0.9)	68	(5.2)	169	(7.1)
Cervix	145	(45.6)	0	(0.0)	0	(0.0)	145	(6.1)
Multiple anatomical sites	43	(13.5)	9	(1.2)	89	(6.7)	141	(5.9)
Other/ Unknown	23	(6.9)	37	(5.0)	116	(8.8)	176	(7.3)
**Probable source of infection**								
Steady partner (solely)	100	(31.4)	95	(12.7)	163	(12.3)	358	(15.0)
Casual partner (solely)	34	(10.7)	305	(40.8)	651	(49.3)	990	(41.5)
Steady and casual partner	31	(9.7)	94	(12.6)	193	(14.6)	318	(13.3)
Being a sex worker	130	(40.9)	10	(1.3)	31	(2.3)	171	(7.2)
Client of sex worker	2	(0.6)	175	(23.4)	10	(0.8)	187	(7.8)
Unknown	21	(6.6)	68	(9.1)	272	(20.6)	361	(15.1)

MSM: Men who have sex with men; MSW: men who have sex exclusively with women.

*128 cases among MSM, 94 among MSW and 13 among females were excluded from the denominator because their HIV status was unknown.

### Clinical characteristics

Almost 40% of cases had a history of STI and this was more common among MSM than among MSW (p < 0.05), whereas there were no significant differences between MSW and women (Table 1). During the study period, the proportion of cases with previous STI increased only among MSM (from 32.4% in 2006 to 65.1% in 2010).

The majority of males sought care because they had symptoms, this not being the case among women. Nevertheless, one in ten MSM were diagnosed with gonorrhoea as a result of screening (Table 1).

The urethra was the most frequent site of infection in male cases followed by the rectum in MSM. Most females had cervical gonorrhoea infection, but in almost 30% *N. gonorrhoeae* was isolated in the pharynx. Multisite infections were more frequent in women and MSM than in MSW (Table 1). A total of 334 MSM (25.3%), 125 MSW (16.7%) and 36 women (11.3%) attributed their infection to oral sex exclusively.

**Figure 2:**
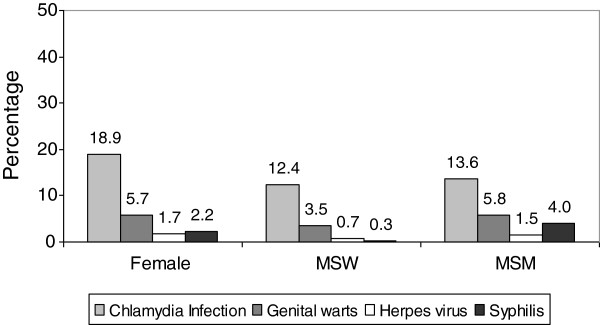
**Gonorrhoea cases diagnosed in 15 STI clinics in Spain from 2006 to 2010.** Concurrent diagnosis of specific STI by sex and transmission route. *MSW: Men who have sex exclusively with women; MSM: Men who have sex with men.

Concurrent diagnoses with other STI (apart from HIV) was quite frequent. Both MSM and heterosexual women had more concurrent diagnoses than MSW (Table 1). *Chlamydia trachomatis* infection and genital warts were the most frequently diagnosed STI in all sex-transmission categories, but MSM had a greater prevalence of concurrent syphilis diagnoses than the other patients (Figure 2).

No case was found to be resistant to ceftriaxone.

### Characteristics related to transmission

Countries other than Spain were reported as the possible place of infection by only 38 patients (1.6%), with no differences between groups.

The majority of males indicated solely “sexual contact with a casual partner” as the likely source of gonorrhoea infection (Table 1). Transactional sex was reported frequently as the likely source of infection among MSW, and these patients were older than their counterparts infected through sex with a steady and/or causal partner (median age: 34 vs. 28 years, p < 0.05); no further differences by region of birth, educational level, HIV status, history of STI or reason for attending the clinic were found among these two groups.

Sex workers were overrepresented among females cases compared to males (Table 1). Most female sex workers (FSW) were foreigners (90.3%), mainly from Latin America and Eastern Europe (68.5% and 15.4% of FSW, respectively), with a median age of 28 years (P_25_-P_75_: 24–34.5) and low educational level (only 26.2% had secondary/university education). Almost one third (30.8%) reported a previous STI, and the most frequent reason for attending the clinic was to undergo periodic screening (53.8%). Pharyngeal gonorrhoea was very common among FSW (53.1%), and 8.5% of them presented multisite infection. Only one FSW was infected with HIV.

A total of 41 men were sex workers (10 MSW and 31 MSM). Their median age was 29.0 years (P_25_-P_75_: 25–34), 46.3% were Latin-Americans, 31.7% Spaniards, 12.2% Eastern Europeans and 9.7% came from other regions. Almost 54% had had STI in the past, and 31.7% were co-infected with HIV. The majority (63.4%) reported symptoms, and the most common locations were the urethra (48.8%) and the rectum (26.8%); one in ten cases had multiple locations.

### HIV Co-infection

Information on HIV status was available for a total of 2150 gonorrhoea cases (90.3% of MSM, 87.4% of MSW and 95.9% of heterosexual females). HIV co-infection was highest among MSM (20.9%) followed by MSW (2.3%) (Table 1). A significant increasing trend in gonorrhoea/HIV co-infection was found among MSM (from 15.9% in 2006 to 27.8% in 2010). In the multivariate analysis factors associated with co-infection were: age older than 35 years, low educational level, origin in Western Europe or Latin-America, being MSM, history of STI, having a concurrent STI, and reporting an HIV infected partner as the source of gonorrhoea infection. On the contrary, women were less likely to be infected with HIV (Table 2).

**Table 2:** Factors related to HIV/gonorrhoea co-infection

**Variables**	**HIV/gonorrhoea co-infection**	
	**AOR**	**95% CI**
**Age group (years) (25–34 years)**		
<25	0.8	0.5-1.2
35-44	1.9	1.3-2.6
≥ 45	1.9	1.1-3.2
**Educational level (Secondary/University education)**		
Illiteracy/Primary education	1.6	1.0-2.4
Unknown	1.2	0.8-1.7
**Region of birth (Spain)**		
Western Europe	2.1	1.2-3.7
Central/Eastern Europe	1.5	0.6-3.8
Latin America	2.0	1.4-2.9
Africa	1.3	0.5-3.5
Other/Unknown	0.6	0.1-2.3
**Sex/Transmission category (MSW)**		
MSM	6.3	3.5-11.4
Female	0.2	0.1-0.8
**Previous STI (No)**		
Yes	3.1	2.1-4.5
**Concurrent STI (No)**		
Yes	1.6	1.2-2.2
**Probable source of gonorrhoea infection (Steady partner)**		
Casual partner (solely)	1.0	0.6-1.6
Steady and casual partner	0.8	0.5-1.5
Being a sex worker	2.0	0.8-4.7
Client of sex worker	0.5	0.1-1.7
**HIV status of sex partner (HIV negative/Unknown)**		
HIV diagnosed	5.9	2.8-12.4
**Year of diagnosis (2006)**		
2007	1.0	0.6-1.8
2008	1.3	0.7-2.2
2009	0.9	0.5-1.4
2010	1.7	1.1-2.8

Reference categories in parentheses.

Multivariate logistic regression analysis.

AOR: Adjusted Odds ratio; CI: confidence interval; MSM: Men who have sex with men; MSW: men who have sex exclusively with women.

Among HIV co-infected people with available information (81.6%) a total of 153 cases (70.2%) were aware of their HIV status before the gonorrhoea diagnosis: 72.6% of MSM (146 of 201 cases), 35.7% of MSW (5 of 14 cases) and 66.7% of women (2 of 3 cases).

## Discussion

This is the first study presenting a description of the epidemiological and clinical characteristics of gonorrhoea cases diagnosed in a broad area in Spain. Its results help to characterise the most-at-risk groups for gonorrhoea infection in the country, and show differences by sex, transmission route and country of birth which should be taken into consideration when implementing control measures.

As shown by the high proportion of cases with previous STI episodes or concurrent STI, and the high HIV prevalence, individuals diagnosed with gonorrhoea in the EPI-ITS network are most likely at high risk of contracting and transmitting gonorrhoea, i.e. they are “core” populations for this infection. This result was to be expected given the characteristics of the participating clinics.

More than half of the cases occurred in MSM whereas, according to the Spanish Survey on Health and Sexual Behaviour (SHSB) (carried out in 2003 in a probability sample of the Spanish population), only 3.9% of men reported having had sex with other men at least once in their lifetime [12]. Even if figures have changed since the survey was performed it is clear that there is an overrepresentation of MSM among gonorrhoea cases in our study, a result common with other studies performed in similar settings both in Spain (Madrid [13] and Barcelona [14]) and elsewhere [15]. HIV prevalence among MSM was very high, 20.9%, although this figure is intermediate between the 31% found in the United Kingdom (UK) [16] and the 11% found in a Stockholm in studies performed in STI clinics [17]. It is worrisome that most HIV-infected MSM were aware of their status before their gonorrhoea diagnosis, and yet they had not used condoms, a finding also described in other studies [17], which highlights the importance of continuous reinforcement of secondary prevention in MSM infected with HIV. The fact that in our study HIV prevalence is increasing only among MSM gonorrhoea cases adds urgency to this recommendation.

The percentage of rectal gonorrhoea among MSM found in our study is lower than reported in STI clinics in Germany and the UK (35% and 31% respectively) [16,18]. Similarly, the proportion pharyngeal location (5.2%) is lower that the figures found in Germany (7.3%) [18], and in the Norwegian surveillance system (7%) [19]. These variations could be due to differences in the study populations but, since both pharyngeal and rectal gonorrhoea are usually asymptomatic, our findings likely reflect low rates of screening in the MSM population, given that in our study the majority of MSM cases were symptomatic, or low rates of comprehensive and adequately tailored testing procedures.

The overrepresentation of foreigners among female patients is the consequence of the high proportion of sex workers among this group, since it is well known than around 90% of FSW in Spain were born out of the country [20]. This is also the most likely explanation for the high proportion of concurrent STI diagnoses found in this and other Spanish studies [21,22].

In spite of the high proportion of concurrent STI diagnoses among FSW with gonorrhoea in our study, HIV prevalence among them is quite low and, if compared with previous studies performed in FSW in Spain, remains stable [21-23]; this could be due, as has been found in other studies [21,22], to high levels of condom use with clients, at least in vaginal sex, and/or to a low HIV prevalence among their clients. Pharyngeal gonorrhoea was very common among FSW in our study (53.1%), a result also found in other studies, which has been attributed to lack of consistent condom use for oral versus vaginal sex [24]. It is also possible that frequent screening in the FSW population has contributed to the high proportion of the pharyngeal location.

Unprotected sex with casual partners was the most important source of infection in male patients. This finding has also been reported in Norway [19]. These results are very important because they reflect a lack of risk perception in men and make it more difficult to conduct partner notification.

An important proportion of MSW in our study contracted gonorrhoea through sexual contact with a FSW. Results from the SHSB showed that 25.4% of men in Spain had had transactional sex in their lifetime, and 5.7% of them had done so in the previous 12 months; at the time of the survey, these were the highest proportions ever described in developed countries [25]. Use of transactional sex was associated, among other factors, with age older than 25, low educational level and foreign origin, which are also common in our population. Interestingly, 95% of men who reported having paid for sex in the last 5 years in the SHSB also reported condom use in their last paid contact, which obviously has not been the case in our study. However, in the SHSB sexual contact was defined as penetrative sex (either vaginal, anal or oral), which is not the case in our study where 35% of male clients of FSW contracted the infection solely through oral sex, a situation in which condom use is less common.

Studies performed in other Western European countries found an important proportion of heterosexual males having been infected with gonorrhoea while abroad [19,26,27]. This is not the case in our study, where in all sex-transmission categories most infections were acquired within the country.

Foreigners are overrepresented in all sex-transmission categories, from a high of 57.9% in women to a low of 28.0% in MSM, comparing to the proportion of foreigners in the general population in Spain (14% in 2010). Latin-Americans are the main immigrant group in all categories, but the second group varies, reflecting different migration patterns among groups [19,28].

This study has limitations. Variables such as educational level and probable source of infection have a substantial proportion of missing data. Furthermore, cases are not representative of all gonorrhoea cases in Spain, therefore the results can only be extrapolated to the setting from which the cases arose and not to the whole Spanish population. However, to our knowledge, most of the STI clinics operating in Spain belong to this network and cases included in this study represent more than one fourth of all gonorrhoea cases declared in Spain from 2006 to 2008.

## Conclusions

This study helps to identify STI core populations for gonorrhoea infection in Spain, and provides important information that should guide policies to be applied in the future to these groups. As is common in most developed countries, MSM emerge as a priority group for the implementation of such policies, in particular, HIV-infected MSM.

## Abbreviations

STI: Sexually transmitted infections; HIV: Human immunodeficiency virus; MSM: Men who have sex with men; MSW: Men who have sex exclusively with women; FSW: Female sex workers; SHSB: Spanish survey on health and sexual behaviour; OR: Odds ratio; CI: Confidence interval.

## Competing interests

The authors declare that they have no competing interests.

## Authors’ contributions

AD supervised field work and data collection, planned and performed the statistical analysis and wrote the first version of the manuscript. CG performed data collection and management, quality control and reviewed all the manuscript drafts. JAV, EF, IS, JB, FG, CC, JLdeM, VE, MLJ, BM, IP, JS, MJB, MA, JMU and STI Study Group: were the clinicians responsible for patient recruitment and follow-up in the participating centres. They all participated in development of the study protocol, collection of epidemiological and clinical data, and critical review of all versions of the manuscript. MD made important contributions to the manuscript and wrote the last version. All authors read and approved the final manuscript.

## Pre-publication history

The pre-publication history for this paper can be accessed here:

http://www.biomedcentral.com/1471-2458/13/1093/prepub

## Members of the STI Study Group

A Diaz, M Diez, C Garriga (Centro Nacional de Epidemiología, Madrid); J Suarez (Centro ETS, Algeciras); JM Ureña, E Castro, V Benavides, M Gómez (Centro de ETS y Orientación Sexual, Granada); B Martínez, MA García, L Godoy (Unidad de Promoción y Apoyo a la Salud, Málaga); I Pueyo, E Ruiz, C Redondo, C Martinez, D Sánchez (Centro de ETS, Sevilla); JA Varela, C López, L Otero (Unidad de ETS, Gijón); ML Junquera, M Cuesta, F Vázquez, F Carreño (Unidad de ETS. Hospital Monte Naranco, Oviedo); L Gil (Centro Atención de ITS (CAITS), Palma de Mallorca); MJ Barberá, E Arellano, P Saladié, P Armengol, M Arando, M Vall, M Gosch, MA Álvarez, A Vives, I Oliveira, G Euba, S Börjesson (Unidad de Infecciones de Transmisión Sexual. Programa especial de enfermedades infecciosas Vall d’Hebron-Drassanes. Hospital Universitario Vall d'Hebron, Barcelona); J Boronat (Unidad ITS, CAP Tarragonès, Tarragona); FJ Bru, C Colomo, A Comunión, R Martín, S Marinero (Programa de Prevención del Sida, Madrid); J del Romero, T Puerta, C Rodríguez, M Vera, B Menéndez, J Ballesteros, P Clavo (Centro Sanitario Sandoval, Madrid); J Belda, E Fernández, T Zafra, S Colomina, E Galán (Centro de Información y Prevención del Sida (CIPS), Alicante); F Gual (Comité de apoyo a trabajadoras del sexo (CATS), Murcia); J Balagué (Unidad ETS, Cartagena); M Cámara, J López de Munain, N Aparicio, A Aizpuru (Unidad ETS.Sº Enfermedades Infeccionas. H. Basurto, Bilbao); V Esteban, R. Cisterna, G Ezpeleta, JA Álava (Consulta ETS. Sº Microbiología Clínica. H. Basurto, Bilbao); I Sanz, X Camino (Consulta ETS. San Sebastián); P Gómez-Pintado (Instituciones Penitenciarias).
